# Impact of perioperative red blood cell transfusion, anemia of cancer and global health status on the prognosis of elderly patients with endometrial and ovarian cancer

**DOI:** 10.3389/fonc.2022.967421

**Published:** 2022-09-16

**Authors:** Katharina Anic, Mona Wanda Schmidt, Marcus Schmidt, Slavomir Krajnak, Amelie Löwe, Valerie Catherine Linz, Roxana Schwab, Wolfgang Weikel, Walburgis Brenner, Christiane Westphalen, René Rissel, Erik Kristoffer Hartmann, Roland Conradi, Annette Hasenburg, Marco Johannes Battista

**Affiliations:** ^1^ Department of Gynecology and Obstetrics, University Medical Center of the Johannes Gutenberg University Mainz, Mainz, Germany; ^2^ Department of Geriatric Medicine, University Medical Center of the Johannes Gutenberg University Mainz, Mainz, Germany; ^3^ Department of Anesthesiology, University Medical Center of the Johannes Gutenberg University Mainz, Mainz, Germany; ^4^ Blood Transfusion Center, University Medical Center of the Johannes Gutenberg University, Mainz, Germany

**Keywords:** prognosis, ovarian cancer, endometrial cancer, transfusion, anemia of cancer, frailty

## Abstract

**Introduction:**

Perioperative red blood cell (RBC) transfusions have been associated with increased morbidity and worse oncological outcome in some solid neoplasms. In order to elucidate whether RBC transfusions themselves, the preoperative anemia of cancer (AOC), or the impaired global health status might explain this impact on patients with endometrial cancer (EC) or ovarian cancer (OC), we performed a retrospective, single-institution cohort study.

**Materials and methods:**

Women older than 60 years with EC or OC were included. The influence of RBC transfusions, AOC, and frailty status determined by the G8 geriatric screening tool (G8 score), as well as the clinical-pathological cancer characteristics on progression-free survival (PFS) and overall survival (OS), was determined by using the Kaplan-Meier method and the Cox regression analyses.

**Results:**

In total, 263 patients with EC (n = 152) and OC (n = 111) were included in the study. Patients with EC receiving RBC transfusions were faced with a significantly shorter 5-year PFS (79.8% vs. 26.0%; p < 0.001) and 5-year OS (82.6% vs. 25.7%; p < 0.001). In multivariable analyses, besides established clinical-pathological cancer characteristics, the RBC transfusions remained the only significant prognostic parameter for PFS (HR: 1.76; 95%-CI [1.01–3.07]) and OS (HR: 2.38; 95%-CI [1.50–3.78]). In OC, the G8 score stratified the cohort in terms of PFS rates (G8-non-frail 53.4% vs. G8-frail 16.7%; p = 0.010) and AOC stratified the cohort for 5-year OS estimates (non-anemic: 36.7% vs. anemic: 10.6%; p = 0.008). Multivariable Cox regression analyses determined the G8 score and FIGO stage as independent prognostic factors in terms of PFS (HR: 2.23; 95%-CI [1.16–4.32] and HR: 6.52; 95%-CI [1.51–28.07], respectively). For OS, only the TNM tumor stage retained independent significance (HR: 3.75; 95%-CI [1.87–7.53]).

**Discussion:**

The results of this trial demonstrate the negative impact of RBC transfusions on the prognosis of patients with EC. Contrastingly, the prognosis of OC is altered by the preoperative global health status rather than AOC or RBC transfusions. In summary, we suggested a cumulatively restrictive transfusion management in G8-non-frail EC patients and postulated a more moderate transfusion management based on the treatment of symptomatic anemia without survival deficits in OC patients.

## Introduction

It is still a matter of debate whether transfusions of red blood cells (RBC) alter the survival prognosis of patients with oncologic diseases or not (1). Furthermore, it remains unclear if RBC transfusions themselves, the underlying anemia of cancer (AOC), or the preoperative global health status influence the outcome in addition to the conventional tumor entity–specific risk factors (2–4).

Despite the effort of restrictive transfusion strategies, the transfusion indication of RBCs is still an essential treatment component especially in almost frail elderly cancer patients, requiring a major tumor reductive debulking surgery (5–7). Perioperative surgical transfusion rates in patients with gynecological malignancies range from 3% (8) to 77% (9–12). Transfusions of allogeneic RBCs can be life-saving in many circumstances and represent one of the main advances of modern medicine, particularly in oncology (13). Overall, RBC transfusions are safer than they have ever been, but there are still significant risks and impaired postoperative outcomes (14–16). Increased cancer recurrence rates and the risk of developing new malignancies are reported in transfused patients affected by solid cancers, mainly in colorectal and gastroesophageal cancer (17–19). The negative effects rely possibly on the transfusion-related immune modulations (20–23) because the activity of natural killer cells and T lymphocytes is reduced by allogeneic RBC transfusions (24). However, these endogenous defense cells are required to prevent quiescent cancer cell dissemination (25). With the exception of cervical carcinoma, the current literature on the effect of RBC transfusions among gynecological cancer patients is limited and partially controversial (14, 26–28).

AOC, one main reason for perioperative RBC transfusions, has been shown to be independently associated with an increased risk of adverse postoperative complications and an increased length of intensive care unit and hospital stay (29). Approximately every second cancer patient scheduled for major oncologic surgery while being anemic even prior to surgery at the time of diagnosis (30). Bleeding, nutritional deficiencies, hemolysis, reduced erythropoietin levels, and inflammation with increased hepcidin activity cause AOC (31). The state of functional iron deficiency as a well-established squeal of chronic anemia is often regarded as a consequence of chronic illness (32). Moreover, perioperative RBC transfusions due to severe AOC have been analyzed as an independent risk of poorer outcomes and adverse events in 941,496 operations from various disciplines (33). Finally, anemic patients undergoing cancer-related therapies suffer more often from advanced oncologic diseases and present more often with a limited global health status (34).

To which extent global health status besides AOC and entity-specific clinical-pathological cancer characteristics might influence the overall outcome depends on the following considerations. Gynecological malignancies mainly affect elderly patients (35, 36). Women with endometrial (EC) or ovarian cancer (OC) are at high risk of RBC transfusions due to a multitude of cancer and treatment-related factors (37, 38). In addition to the high median age of approximately 68 years at diagnosis (39) increased age is often associated with more aggressive and advanced diseases and requires extended, curatively intended surgical procedures (40, 41). However, the population older than 65 years of age is less likely to be treated in accordance with the recommendations of internal guidelines resulting in an overall worse outcome in elderly cancer patients (42, 43). Advanced diseases are often associated with an impaired individual global health status and malnourishment in the elderly population. This causes a not negligible impact on the decision to perform extensive surgical cytoreduction, possibly with multi-visceral resections to achieve complete macroscopic tumor resection (44–46). The “phenotype of frailty” can be defined as a multidimensional aging-related clinical syndrome of decreased homeostatic reserves and function due to various organ systems which could form a non-standardized definition of the global health status, especially in elderly cancer patients (47, 48). Frail patients are characterized by vulnerability to adverse health outcomes as well as the combination of dysregulation across various physiologic and molecular pathways (49). Various global health assessment tools exist in order to detect preoperative frailty (48, 50, 51). The G8 geriatric screening tool (G8 **s**core) is one of the most commonly used rapid geriatric screening questionnaires (52, 53). The G8 **s**core has been evaluated especially in oncological-surgical disciplines because of its main focus on nutrition, mobility, and comorbidities (54, 55).

We try to elucidate the impact of RBC transfusions, AOC, and the pre-surgical global health status on the outcome of elderly patients with EC and OC in a retrospective, single-institution cohort study.

## Materials and methods

### Study population

This retrospective cohort analysis reports data from women older than 60 years of age surgically treated at the University Medical Center Mainz – Johannes-Gutenberg University Mainz, Germany, between January 2008 and December 2019. Patients at all stages of EC and OC who were being operated on with curative intent were screened and included if they fulfilled the following criteria: 1) Patients with EC receiving a standardized primary staging operation including hysterectomy and bilateral salpingo-oophorectomy, with or without pelvic and para-aortic lymph node resection, depending on tumor stage, histological grade of differentiation, and histological subtype. 2) Patients with OC who underwent primary or interval tumor debulking surgery with maximal surgical effort. 3) Patients for whom the determination of the G8 **s**core was possible. 4) OC and EC patients for whom complete follow-up information was available.

### Data collection

General patient information was gathered from our electronic hospital database SAP (Walldorf, Germany, 1972) and the archives, including clinical-pathological cancer characteristics such as tumor stage [TNM and FIGO (International Federation of Gynecology and Obstetrics) classification system (56) and histological grade] and surgical parameters (e.g., amount of blood transfusions, blood loss, or operating time). Perioperative RBC transfusions were defined as any transfusions within 24 h preoperatively, during surgery, or 24 h after surgery. Transfusions due to surgical bleedings within operative revisions were not included in the final evaluation. Preoperative hemoglobin was taken from the electronic patient record. The cutoff for preoperative anemia was chosen with a hemoglobin <12 g/dl, according to the definition of the World Health Organization for women (57). The patients’ preoperative global health status was retrospectively assessed with the G8 **s**core based on the routine pre-surgical patient evaluation. This process has previously been described elsewhere (54, 58). Long-term follow-up including progression-free survival (PFS) and overall survival (OS) was performed by telephone calls, written inquiries to the patients or their physicians, and by checking the patient clinical records up to February 2021.

### Clinical-pathological cancer characteristics and intraoperative treatment parameters

Clinical-pathological cancer characteristics were collected from patients’ charts. Standardized operation reports were reviewed to extract the information on intraoperative blood loss, cut-seam time, and surgical radicality using the surgical complexity score (SCS). SCS was established by Aletti et al. in order to categorize the maximal surgical effort into a low, intermediate, and high level of complexity (44).

### Frailty assessment – G8 geriatric screening tool

The G8 geriatric screening tool (G8 **s**core) established by Bellera et al. in 2012 was chosen as one of the most frequently used frailty evaluation tools recommended by the International Society of Geriatric Oncology (SIOG) to characterize the preoperative global health status (53). As a simple, time-saving, and reproducible questionnaire, the G8 **s**core consists of seven items from the Mini Nutritional Assessment (MNA) questionnaire with predefined answer options in combination with the chronological age (59–61). The several items assessed in the G8 **s**core are routinely recorded through a standardized health status self-assessment questionnaire in accordance with the MNA as a standard procedure during the pre-surgical consultation. Adding the missing item “biological-calendar age” allows us to calculate the G8 **s**core retrospectively for each patient. The main categories arise from physical performance status and mobility, nutrition, and comorbidities in combination with polypharmacy. The scoring system ranges from 17 points (not impaired at all: G8-non-frail) to a minimum of 0 points (heavily impaired: G8-frail) using the validated cutoff value of ≤14 points as an indicator of frailty (52). In various surgical disciplines, the G8 **s**core is validated to preoperatively identify frail patients, who could benefit from a full comprehensive geriatric assessment (CGA) after a two-step evaluation before major surgery (62).

### Statistical analyses

The manuscript was written following the STrengthening the Reporting of Observational Studies in Epidemiology (STROBE)—a cohort checklist of the Enhancing the QUAlity and Transparency Of health Research (EQUATOR) network reporting guidelines (63). Statistical analyses were performed with the use of the SPSS statistical software program, version 27.0.1 (SPSS Inc, Chicago, IL, U.S.A.). Patients’ characteristics are given in absolute and relative frequencies (categorical data). The frequency of distribution of categorical variables was compared with Fisher’s exact test. For continuous data, normal distribution was explored using the Shapiro-Wilk test. Between-group differences (e.g., transfused vs. non-transfused or “G8-frail” vs. “G8-non-frail”) were explored using either the Mann-Whitney U-test or a t-test to evaluate for significant differences. A *post-hoc* power calculation was used to underline the sufficient number of subjects using an alpha error rate of 0.05. The Cox proportional hazard regression model was used to determine the prognostic influence of preoperative hemoglobin results, perioperative RBC transfusions, and the preoperative frailty status assessed by the G8 **s**core. Furthermore, established entity-specific clinical-pathological cancer characteristics such as tumor stage at diagnosis (according to the FIGO stage), histological grade of differentiation, and histological subtype, as well as surgical parameters, were included in the Cox regression analyses. Firstly, a univariable Cox regression analysis for every single variable was performed. Secondly, variables with a p-value < 0.10 were included in the multivariable Cox regression analyses with a variable selection *via* backward elimination. In the Cox regression model, hazard ratios (HRs) with their 95%-confidence interval (95%-CI) and p-values were used. Kaplan-Meier estimates were used to describe PFS and OS after 5 years. Time points in months were the date of diagnosis which resulted in the operation date up to death (or recurrence) or last follow-up. Consequentially, PFS was defined as the length of time after the primary operation that a patient lives without a relapse. In the case of residual tumor burden, PFS was defined as the time after primary surgery until clinical or radiological progression of the disease was found. OS was measured from the date of operation to the date of death or last follow-up. The log-rank test was used to compare the survival curves. All tests were two-sided and a p-value of < 0.05 was considered exploratory, because no correction for multiple testing was performed.

## Results

### Endometrial cancer

#### Clinical-pathological cancer characteristics

**Figure 1:**
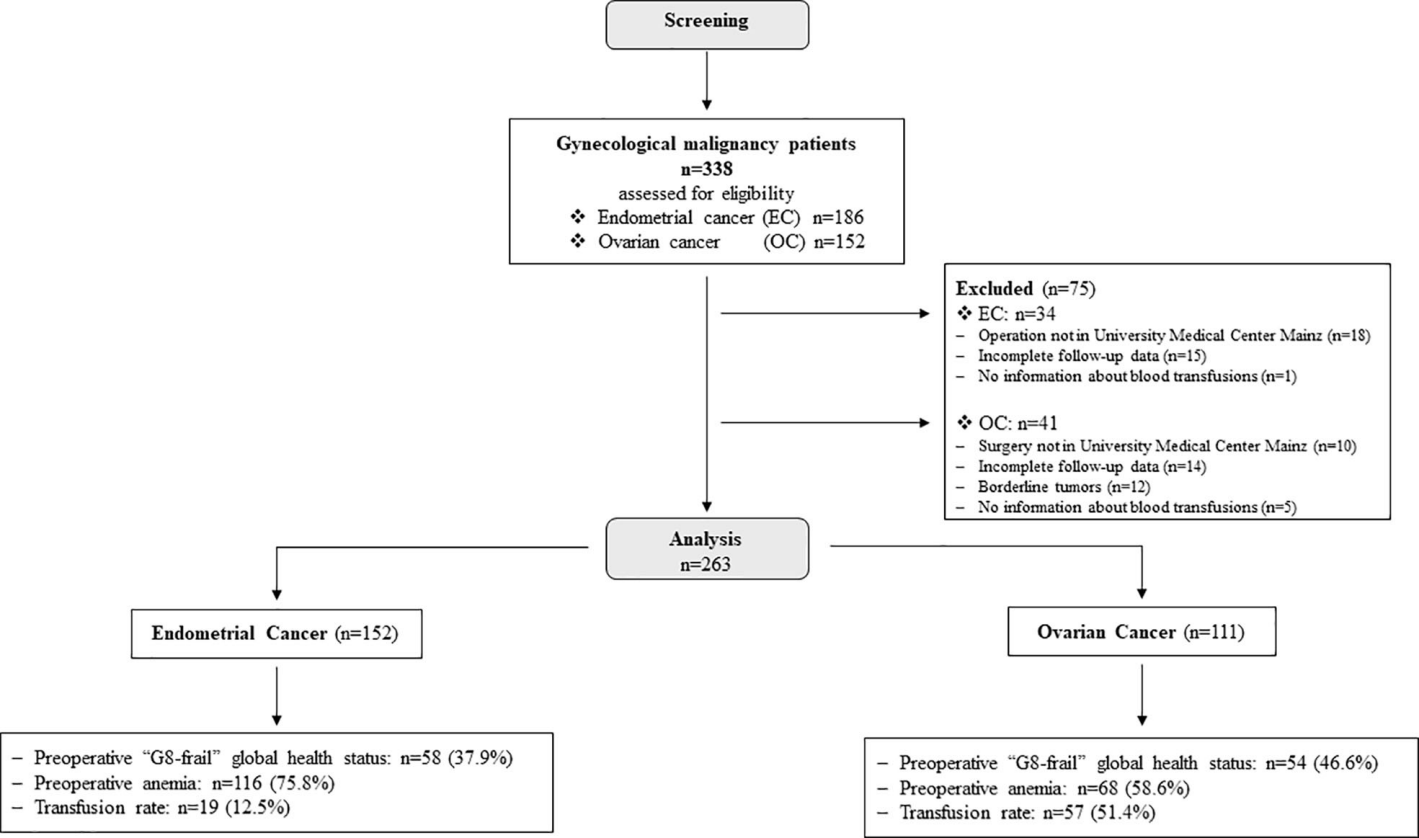
Consort-Statement.

A total of 338 patients were screened and, finally, 263 women entered the study. Out of them, 152 (57.8%) patients suffered from EC (**Figure 1**). The median follow-up time in this sub-cohort was 31.0 [8.0–68.5] months. The mean age of the study population did not differ between the two cancer entities (EC: 71.0 ± 7.4 years and OC: 70.9 ± 5.9 years) (**Table 1**).

**Table 1:** Patients’-characteristics gynaecological malignancies.

			Endometrial Cancer (EC)			Ovarian Cancer (OC)			
Parameter n (%) (+/- SD)			total n=152	transfused n=19	non- transfused n=133	total n=111		transfused n=57	non- transfused n=54
**Mean age** [years]			71.0 (+/- 7.4)			70.9 (+/- 5.9)			
**Clinical-pathological cancer characteristics**									
**Tumor Stage** (FIGO-Stage)			**p<0.001**			p=0.483			
**I**			123 (80.9)	11 (57.9)	111 (84.1)		13 (11.2)	4 (7.0)	9 (16.7)
		**Ia**	70 (46.1)			**Ia**	8 (6.9)		
		**Ib**	53 (34.9)			**Ib**	1 (0.9)		
						Ic*	4 (3.4)		
**II**			7 (4.6)	0 (0.0)	7 (5.3)		6 (5.2)	3 (5.3)	3 (5.6)
						**IIa**	2 (1.7)		
						**IIb**	4 (3.4)		
**III**			12 (7.9)	2 (10.5)	10 (7.6)		74 (63.8)	36 (63.2)	35 (64.8)
		**IIIa**	2 (1.3)			**IIIa1**	5 (4.3)		
		**IIIb**	3 (2.0)			**IIIa2**	0 (0.0)		
		**IIIc1**	5 (3.3)			**IIIb**	13 (11.2)		
		**IIIc2**	2 (1.3)			**IIIc**	56 (48.3)		
**IV**			10 (6.6)	6 (31.6)	4 (3.0)	17 (14.7)		10 (17.5)	7 (13.0)
		**IVa**	3 (2.0)			2 (1.7)			
		**IVb**	7 (4.6)			15 (12.9)			
**Histological Subtype**			**p=0.005**			p=0.824			
	**Endo-metrioid**		130 (85.0)	12 (63.2)	117 (88.0)	**Serous**	86 (74.1)	18 (31.6)	16 (29.6)
						low grade	5 (4.3)		
						high grade	81 (69.8)		
	**Others**		23 (15.0)	7 (36.8)	16 (12.0)	**Others**	30 (25.9)	39 (68.4)	38 (70.4)
	(serous, squamous, mucinous,)					(endometrioid, mucinous, clear cell)			
**Histological grade of differentiation**			p=0.774			p=0.560			
**G1**			75 (49.0)	7 (36.8)	67 (50.4)	6 (5.2)		2 (3.5)	4 (7.4)
**G2**			46 (30.1)	6 (31.6)	40 (30.1)	21 (18.1)		9 (15.8)	11 (20.4)
**G3**			30 (19.6)	4 (21.1)	26 (19.5)	87 (75.0)		44 (77.2)	39 (72.2)
**Red blood cell transfusion management**									
**Timing of transfusion**									
preoperative intraoperative postoperative			4 (21.1) 12 (63.2) 3 (15.8)			6 (10.5) 42 (73.7) 9 (15.8)			
**Number of transfusions**									
≤ 5> 5			16 (84.2)3 (15.8)			38 (66.7)19 (33.3)			
**Preoperative anemia of cancer**									
**[g/dl]**			**p<0.001**			**p=0.030**			
Haemoglobin < 12			35 (23.3)	14 (77.8)	21 (15.9)	40 (37.4)		26 (47.3)	14 (26.9)
Haemoglobin > 12			115 (76.7)	4 (22.2)	111 (84.1)	67 (62.6)		29 (52.7)	38 (73.1)
**Global health status**									
**G8 Score**			**p<0.001**			**p=0.031**			
G8-frail G8-non-frail			58 (38.9) 91 (61.1)	15 (83.3) 3 (16.7)	43 (33.1) 87 (66.9)	51 (48.1) 55 (51.9)		32 (58.2) 23 (41.8)	19 (37.3) 32 (62.7)
**Surgical treatment parameters**									
Postoperative Residual tumor burden			p=0.150			**p=0.017**			
None Present Unknown			144 (94.7) 6 (3.9) 2 (1.3)	16 (84.2) 2 (10.5) 1 (5.3)	127 (95.5) 4 (3.0) 2 (1.5)	67 (58.3) 48 (41.7) 0 (0.0)		27 (47.4) 30 (52.6) 0 (0.0)	37 (68.5) 16 (29.6) 0 (0.0)
SCS – Surgical Complexity Score			n.a.			*p=0.062*			
SCS 1 SCS 2 SCS 3						37 (33.3) 53 (47.7) 21 (18.0)		16 (28.1) 26 (45.6) 15 (26.3)	21 (39.6) 27 (50.9) 5 (9.4)
Completeness of systemic therapy			p=0.425			p=0.841			
No Yes			2 (14.3) 12 (85.7)	0 (0.0) 3 (25.0)	2 (100.0) 9 (75.0)	13 (16.0) 68 (84.0)		6 (16.2) 31 (83.8)	7 (17.9) 32 (82.1)

EC, endometrial cancer; FIGO, International Federation of Gynecology and Obstetrics; G, histological grade of differentiation; G8 Score, G8 geriatric Screening tool; G8 frail, G8 geriatric Screening tool > 14 points; G8 non-frail, G8 geriatric Screening tool ≤ 14 points; OC, ovarian cancer; SD, standard deviation; SCS, Surgical Complexity Score.

n.a.: not applicable, n: number of patients.

*if the number of cases is small, the subdivision into IC1, IC2 and IC3 is waived.

**bold written words**: analyzed main categories.

**bold written numbers**: statistically significant results (p<0.05); italic written numbers: clinically relevant results (p<0.1).

A higher FIGO **s**tage of EC required significantly more RBC transfusions than lower FIGO **s**tages (transfused: FIGO III–IV: 42.1% vs. non-transfused FIGO III–IV: 9.6%; transfused: FIGO I–II: 57.9% vs. non-transfused FIGO I–II: 89.4%; p < 0.001). By histologic subtype, significantly, more women with endometrioid cancers were recorded in the EC-transfused cohort (63.2% vs. 36.8%; p = 0.005). The histological grade of differentiation was not associated with RBC transfusions (p = 0.774).

#### Surgical treatment parameters

The transfused EC patients (84.2%) received up to five RBC transfusions, mostly during surgical procedures (63.2%). AOC was associated with RBC transfusion indication (77.8% vs. 22.2%; p < 0.001). The mean cut-seam time was 142.4 min (± 82.2 min) with a mean intraoperative blood loss of 229.5 ml (± 422.8 ml). Forty-nine patients (32.0%) received laparoscopic surgery, 72 (49.7%) received open surgery, and the remaining 29 (18.3%) received vaginal surgery. In total, four operative revisions were necessary, two due to an incarcerated intestinal loop and two due to a subsequent postoperative hemorrhage. In both circumstances, one of two patients received RBC transfusions. A total of 144 EC patients (94.7%) were operated on without any residual tumor burden. Frail patients were operated on with the same surgical radicality as non-frail patients (data not shown). In total, 14 (9.2%) patients received adjuvant chemotherapy, which was completed in 12 (85.7%) patients. Moreover, 67 and (43.9%) women received adjuvant radiotherapy [61 (39.9%) brachytherapy, 5 (3.3%) percutaneous radiation, and 1 (0.7%) local radiation]. The indication was stage-appropriated in 137 (89.5%) cases.

#### Global health status

Preoperative global health status evaluation with the G8 **s**core allocated 58 (38.9%) patients in the G8-frail and the remaining 91 (61.1%) patients in the G8-non-frail cohort. Frail patients received significantly more RBC transfusions than G8-non-frail patients (83.3% vs. 16.7%; p < 0.001). G8-frail patients receiving RBC transfusions were faced with the lowest survival rates compared to their non-frail and non-transfused counterparts (**Table 3**). The impact of intraoperative RBC transfusions on the survival rates was more pronounced than the influence of the preoperative frailty status (5-year OS: transfused: 25.7% vs. G8-frail: 49.7%).

#### Prognosis

**Figure 2:**
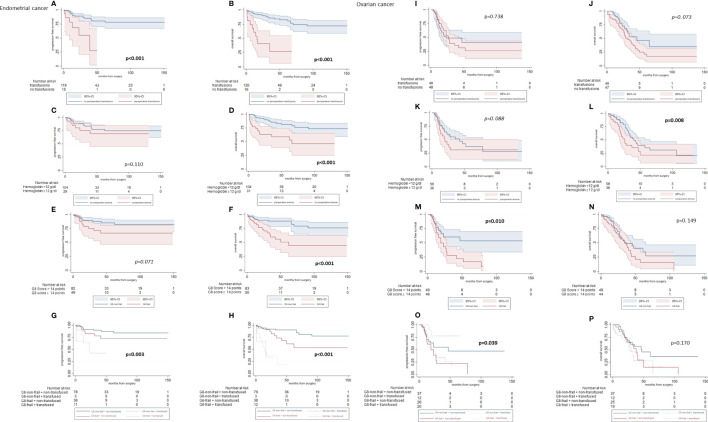
Statistical survival analyses according to the transfusion status of endometrial and ovarian cancer patients. **(A–H)** Endometrial cancer: Kaplan Meier curves. **(I–P)** Ovarian cancer: Kaplan Meier curves.

Kaplan-Meier plots yielded 5-year statistically different OS rates for RBC transfusions (non-transfused: 82.6% vs. transfused: 25.7%; p < 0.001), AOC (non-anemic: 81.2% vs. anemic: 57.1%; p < 0.001), and global health status (G8-non-frail: 88.2% vs. G8-frail: 49.7%; p < 0.001) (**Table 2**). Overall, frail and transfused patients had the worst prognosis in the EC cohort (**Figure 2**). In the univariable Cox regression analysis, FIGO **s**tage, histological grade of differentiation, postoperative residual tumor burden, and RBC transfusions, as well as preoperative frailty status, were associated with decreased survival rates for both, 5-year PFS and 5-year OS (all p-values < 0.05) (**Table 3**). In the multivariable analyses, besides selected clinical-pathological cancer characteristics (FIGO **s**tage and histological grade of differentiation), only RBC transfusions retained their independent significance for both 5-year PFS and 5-year OS (all p-values < 0.05). However, AOC and G8-Status were not independently associated with PFS and OS (all p-values > 0.05).

**Table 2:** Estimated 5-year survival rates by Kaplan-Meier method.

	Endometrial Cancer			Ovarian Cancer		
	n (%)	PFS after 5 years [%], *p value*	OS after 5 years [%], *p value*	n (%)	PFS after 5 years [%], *p value*	OS after 5 years [%], *p value*
**Red blood cell (RBC) transfusions** non-transfused transfused	152 133 (87.5) 19 (12.5)	**<0.001** 79.8 26.0	**<0.001** 82.6 25.7	111 54 (48.6) 57 (51.4)	0.738 40.8 26.0	*0.073* 46.3 20.8
**Preoperative anemia of cancer (AOC)** non-anemic anemic	151 116 (76.8) 35 (23.2)	0.110 77.2 65.0	**<0.001** 81.2 57.1	110 72 (65.5) 38 (34.5)	*0.088* 39.5 26.9	**0.008** 36.7 10.6
**G8 geriatric Screening tool (G8 Score)** G8-non-frail G8-frail	150 92 (61.3) 58 (38.7)	*0.071* 82.1 65.4	**<0.001** 88.2 49.7	110 56 (50.9) 54 (49.1)	**0.010** 53.4 16.7	0.149 40.5 15.3
**Frail – RBC transfusions** G8-non-frail + non-transfused G8-non-frail + transfused G8-frail + non-transfused G8-frail + transfused	148 87 3 43 15	**0.003** 86.0 66.7 72.4 39.2	**<0.001** 90.2 33.3 61.3 17.3	99 38 (38.4) 13 (13.1) 27 (27.3) 21 (21.2)	**0.039** 47.2 77.8 22.3 17.0	0.170 45.5 27.8 14.6 14.6

AOC, anemia of cancer; OS, overall survival; PFS, progression free survival; RBC, red blood cell.

n = number of patients; G8 frail: G8 geriatric Screening tool > 14 points, G8 non-frail: G8 geriatric Screening tool ≤ 14 points

**bold written words**: analyzed main categories;

**bold written numbers**: statistically significant results (p<0.05); italic written numbers: clinically relevant results (p<0.1).

**Table 3:** Uni- and multivariable Cox-regression analyses for survival in patients with gynecological malignancies.

	Endometrial Cancer						Ovarian Cancer					
univariable	PFS			OS			PFS			OS		
	HR	95%- CI	p-value	HR	95%- CI	p-value	HR	95%- CI	p-value	HR	95%- CI	p-value
TNM-Tumor Stage	1.48	0.89-2.47	0.134	2.21	1.56-3.15	**<0.001**	2.56	1.48-4.42	**0.001**	3.41	1.86-6.22	**<0.001**
FIGO-Stage	1.87	1.34-2.61	**<0.001**	2.25	1.69-2.99	**<0.001**	6.21	1.91-20.18	**0.002**	1.96	1.32-2.89	**0.001**
Histological subtype	0.45	0.18-1.12	*0.087*	0.34	0.16-0.73	**0.006**	1.52	0.80-2.86	0.200	1.77	0.93-3.36	*0.083*
Histological grade of differentiation	1.93	1.19-3.12	**0.008**	2.26	1.44-3.55	**<0.001**	1.59	0.91-2.80	0.104	1.71	0.98-2.97	*0.057*
Postoperative residual tumor burden	2.22	1.06-4.65	**0.034**	2.95	1.71-5.08	**<0.001**	2.07	1.17-3.67	**0.012**	3.03	1.70-5.41	**<0.001**
SCS – Surgical Complexity Score	**-**						1.46	0.96-2.22	*0.078*	1.509	1.01-2.26	**0.045**
Completeness of systemic therapy	**-**						2.06	0.87-4.85	*0.071*	0.984	0.49-1.96	0.963
RBC transfusions	4.97	2.03-12.18	**<0.001**	7.48	3.48-16.08	**<0.001**	1.10	0.62-1.98	0.743	1.66	0.95-2.93	*0.078*
Preoperative anemia of cancer (AOC)	0.53	0.24-1.18	0.118	0.29	0.15-0.58	**0.001**	0.58	0.31-1.10	*0.097*	0.46	0.26-0.83	**0.010**
G8 geriatric Screening tool (G8 Score)	2.29	1.04-5.02	**0.040**	3.55	1.73-7.26	**0.001**	2.14	1.17-3.92	**0.014**	1.49	0.86-2.57	0.154
**multivariable**												
TNM-Tumor Stage	–			0.92	0.54-1.56	0.759	1.25	0.45-3.47	0.671	3.75	1.87-7.53	**<0.001**
FIGO-Stage	1.25	1.06-1.46	**0.007**	1.30	1.13-1.49	**<0.001**	6.52	1.51-28.07	**0.012**	1.10	0.12-9.89	0.932
Histological Subtype	3.17	0.82-12.33	*0.096*	3.83	1.15-12.74	**0.029**	**-**			1.38	0.70-2.73	0.351
Histological grade of differentiation	2.25	1.22-4.14	**0.009**	2.11	1.23-3.61	**0.007**	**-**			1.45	0.72-2.93	0.299
Postoperative residual tumor burden	1.29	0.51-3.25	0.586	1.29	0.57-2.91	0.543	1.35	0.70-2.63	0.375	0.83	0.42-1.67	0.605
SCS	–						1.08	0.65-1.79	0.778	1.03	0.64-1.65	0.899
Completeness of systemic therapy	–						1.81	0.68-4.80	0.231	-		
RBC transfusions	1.76	1.01-3.07	**0.046**	2.38	1.50-3.78	**<0.001**	**-**			0.85	0.43-1.68	0.643
Preoperative AOC	–			1.07	0.50-2.30	0.860	1.18	0.59-2.38	0.644	1.70	0.92-3.15	*0.090*
G8 Score	1.34	0.53-3.36	0.533	2.02	0.87-4.67	0.101	2.23	1.16-4.32	**0.017**	-		

95%-CI, confidence interval; FIGO, International Federation of Gynecology and Obstetrics; G8 Score, G8 geriatric Screening tool; HR, hazard ratio; OS, overall survival; PFS, progression free Survival; RBC, red blood cell; SCS, Surgical Complexity Score.

n = number of patients.

**bold written words**: analyzed main categories;.

**bold written numbers**: statistically significant results (p<0.05); italic written numbers: clinically relevant results (p<0.1).

### Ovarian cancer

#### Clinical-pathological cancer characteristics

A total of 111 patients with OC (43.1%) met the inclusion criteria (**Figure 1**), and 51.4% of patients received RBC transfusions (**Table 1**). The conventional clinical-pathological cancer characteristics such as FIGO **s**tage, histological grade of differentiation, and histological subtype did not differ between the transfused and not-transfused cohort. The median follow-up time was 26.0 [12.0–39.0] months.

#### Surgical treatment parameters

AOC was diagnosed in 37.4% of OC patients, and 47.3% of patients receiving RBC transfusions suffered from AOC. Otherwise, solely 26.9% of women with AOC did not receive RBC transfusions and 73.1% of non-anemic patients did not receive RBC transfusions (p = 0.030). The mean operation time was 260.0 min (± 122.7 min) with a mean blood loss of 1,015.32 ml (± 1468.82 ml). In total, 82 (78.1%) patients were treated with primary debulking surgery, and 23 (21.9%) patients received an interval debulking surgery. Nineteen (17.1%) operative revisions were performed due to deep and superficial wound dehiscence in eight (38.1%) cases, intestinal complications as anastomosis insufficiencies or peritonitis in seven (36.8%) cases, and surgical bleeding in four (21.1%) cases. Fourteen of these 19 (73.7%) patients required RBC transfusions. No residual tumor burden was achieved in 67 (58.3%) patients. RBC transfusions were associated with surgical radicality determined by the SCS (p = 0.062) and postoperative residual tumor burden (p = 0.017). Similar to the EC group, the OC patients mostly received five or fewer (66.7%) RBS intraoperatively (73.7%).

#### Global health status

The G8 **s**core allocated 51 (48.1%) patients to the G8-frail group and 55 (51.9%) patients to the G8-non-frail group. Significantly, more G8-frail OC patients were transfused than G8-non-frail (58.2% vs. 41.8%; p = 0.031). Frail patients were operated on with the same surgical intent as non-frail patients (data not shown). G8-frail patients were faced with an impaired PFS compared to their G8-non-frail counterparts (53.4% vs. 16.7%; p = 0.010) (**Figure 2**). No significant survival difference was recognized in terms of 5-year OS (G8-non-frail: 40.5% vs. G8-frail: 15.3%, p = 0.149) (**Table 2**).

#### Prognosis

RBC transfusions did not influence the prognosis in OC patients in terms of PFS (40.8% vs. 26.0%, p = 0.738) and OS (46.3% vs. 20.8%, p=0.073). The *post-hoc* power calculation for the dichotomous endpoint of the two independent sample studies was 82.1%. Anemic patients were faced with a worse outcome in terms of OS when compared with non-anemic patients (10.6% vs. 36.7%; p = 0.008) but not in terms of PFS (26.9% vs. 39.5%; p = 0.088). The univariable Cox regression analyses were shown in **Table 3**. In multivariable Cox regression analyses, solely the FIGO **s**tage and G8 **s**core retained independent significance as prognostic factors for PFS (HR: 6.52; 95%-CI [1.51–28.07] and HR: 2.23; 95%-CI [1.16–4.32], respectively). AOC missed the statistical level of significance (HR: 1.18; 95%-CI [0.59–2.38]). In terms of OS, the TNM tumor stage achieved statistical and AOC clinical significance (HR: 3.75; 95%-CI [1.87–7.53] and HR: 1.70; 95%-CI [0.92–3.15], respectively) in multivariable analyses.

## Discussion

To the best of our knowledge, we here reported for the first time the relationship between RBC transfusions, AOC, and global health status with their possible impact on prognosis for cancer patients and try to elucidate the real source of the fundamental reason for performing a Cox regression analyses. In EC, RBC transfusions and selected clinical-pathological cancer characteristics impaired PFS and OS. In OC, the FIGO **s**tage and frailty status seemed to be the most important prognostic factors for PFS followed by the TNM tumor stage and AOC in terms of OS.

The correlation between perioperative RBC transfusions and postoperative outcome on survival seemed to be entity-specific and was discussed controversially in the current literature. Bogani and colleagues retrospectively examined 275 patients with locally advanced cervical cancer scheduled to undergo neoadjuvant chemotherapy plus radical surgery (15). They reported no association between RBC transfusions and worse disease-specific survival (DSS) (HR: 2.71; 95%-CI [0.91–8.03]). Contrastingly, in gastrointestinal tumor surgery, e.g., esophageal cancer resections, RBC transfusions have been correlated in 568 esophagectomies with a significantly poorer short- and long-term survival (PFS: HR:1.8: 95%-CI: [1.2–2.5] and OS: HR: 2.2; 95%-CI [1.5–3.2], respectively) (17). Although we recorded a restrictive blood management with a transfusion rate of only 12.5% in the EC cohort, RBC transfusions retained its independent significance according to poorer 5-year PFS and 5-year OS. Uccella and colleagues similarly proved the association between RBC transfusions and a higher risk of recurrence in 331 women with EC (27). They hypothesized that RBC transfusions potentially promoted the intraabdominal spread of neoplastic cells due to the transitory perioperative immunodepression (64, 65). For elderly patients with OC, our data could not show an independent impact of the RBC transfusions but for AOC and clinical-pathological cancer characteristics on prognosis. Our results were in line with the findings of Warner and colleagues. They refuted an independent influence of RBC transfusions on survival in women with epithelial OC even if RBC transfusions were significantly associated with age, advanced stages of diseases, and higher surgical complexity (9). In contrast to these findings, Zhang and colleagues postulated a significant deleterious effect on cancer survival related to RBC transfusions in their retrospective study (66). Furthermore, De Oliveira and colleagues were able to demonstrate an association between advanced OC and RBC transfusions in their retrospective cohort investigation (67), although the radicality of tumor debulking surgery, as well as residual tumor burden as validated independent predictors of poor oncological outcomes, was not considered as a potential variable influencing the outcome in that study.

To clarify the indications of RBC transfusions in the perioperative setting, one might address the rule of AOC (2). Our results suggested a poorer 5-year OS for pre-surgical anemic EC and OC patients in Kaplan-Meier plots. In the multivariable Cox regression analyses, an independent prognostic influence was solely demonstrated in OC in terms of OS. Our results were comparable with the results of a recently published prospective trial with 192 patients by Chen and colleagues. “Specifically in obese, nondiabetic, elder, advanced stage but having relatively good performance status patients” a low preoperative hematocrit, lower than 35%, was a valuable predictor of OC women’s poor prognoses (68). Contrastingly, Abu-Zaid and colleagues were not able to determine an independent prognostic association between AOC and OS in endometrioid-type EC in a retrospective cross-sectional study (69). Moreover, Abu-Zaid et al. demonstrated that poorer survival outcomes were predicted by preoperative AOC in patients with exclusively advanced FIGO **s**tage EC in their subsequent systematic review and meta-analysis from 2021 (70). Possibly, our contrasting findings might be explained by the lower rate of 14.5% suffering from an advanced EC.

For the preoperative global health status in cancer patients, our study group could demonstrate the independent impact on postoperative prognosis (54, 55, 58). Consecutively, the frailty status was seen as a potential confounder in this study and was included in the multivariate analyses.

Unfortunately, the important question to be answered remains open: how to manage AOC in the elderly partially frail patients with EC or OC before major surgery? From the presented results, one might conclude to clarify AOC and determine global health status with validated geriatric screening tools, which was beneficial especially in OC patients. These mainly preoperatively detectable parameters seemed to give important insights into the patients’ prognosis regardless of the administration of RBC transfusions. Especially in older women with OC, a multilayered and interdisciplinary diagnostic or rehabilitation program might be helpful to enable an individual therapy concept mainly in frail patients, as G8-frail patients were faced with a poor prognosis irrespectively of maximal surgical effort (58). A moderate transfusion management, based on the fact that RBC transfusions in symptomatic anemic OC patients, was not associated with an overall poorer outcome. However, a restrictive perioperative RBC transfusion management seemed to be much more important in patients with EC, most likely due to the lower surgical radicality and the overall better general condition. Boone and colleagues postulated a strongly restrictive transfusion policy in gynecologic oncology (71). In their retrospective chart review, they examined 582 women, 55.9 years of mean age, with various gynecological malignancies, receiving a total of 2,276 blood transfusions. Their hypothesis was based on the findings that solely women with symptomatic anemia with hemoglobin results <7 g/dl or an intraoperative blood loss of more than 1,500 ml should be transfused, without increased postoperative morbidity, concerning infections, thrombotic events, or mortality. Moreover, the American College of Surgeons National Surgical Quality Improvement Program (NSQIP) published data from 8,519 women, gynecologic surgically treated between 2010 and 2012, in a large-scale multi-institutional dataset. They reported an RBC transfusion rate of 13.8% with a significant higher transfusion-related composite morbidity (odds ratio (OR) = 1.85; 95%-CI [1.5–2.24]), including surgical site infections (OR=1.80, 95%-CI [1.39–2.35]) and length of hospital stay (non-transfused: 3.02 vs. transfused 7.17 days, p=<0.001) (72). However, Boureau and de Decker were able to examine that liberal transfusion strategies could show lower mortality rates, especially in a surgical ward in elderly cancer patients (73). They postulated that transfusion decisions should be based on “benefit/risk balance taking into account patients’ symptoms”. Further trials reviewed evidence-based indications for RBC transfusions, almost in conservative treated patient cohorts, and resumed the potential risks and complications of blood interventions (3). Considering the RBC transfusion management in gynecologic oncologic surgery, most of the current literature had been limited by small sample sizes of about 150 patients (38, 74). In addition, solely univariable analyses showed significant differences between the transfused and non-transfused study sub-cohort (16). If multivariable regression models were used to elucidate the possible effect of RBC transfusions on survival, key perioperative parameters such as surgical complexity or radicality as well as intra-operative blood loss and preoperative hemoglobin level were not included (27, 75, 76). We tried to overcome some of these limitations and demonstrated an independent impact of RBC transfusions and selected clinical-pathological cancer characteristics on the prognosis of patients with EC but not in OC.

By analyzing the impact of RBC transfusions, AOC, and the global health status in the context of known influential clinical-pathological cancer characteristics on the prognosis of EC and OC, this study tried to elucidate the underlying causative mechanism of these intertwined conditions causing a poorer prognosis and went beyond the pure description of an association between RBC transfusions in patients with EC and OC. Limitations arise from the retrospective nature of the data analyses limiting the generalizability of our findings. This might be relevant, particularly in terms of incomplete follow-up, which was successfully reduced to a minimum of 34 EC and 41 OC patients by reaching out to patients and physicians through different channels of communication and an extensive review of clinical records. Nevertheless, the large number of considered entity-specific clinical-pathological prognostic parameters as well as the multidimensional nature of the included patients regarded the frailty status and surgical aspects besides current clinical risk factors strengthen the validity of our results. Additionally, this work was carried out in a single institution. In contrast, the benefit of this single-center trial was the depth of data available, allowing for the analysis of possible confounding variables related to outcomes. Moreover, selection bias that could arise from the decision to transfuse was subjective and some practitioners might have been more liberal with transfusions than others, although the overall rate of perioperative RBC transfusions in this cohort was moderate at 28.3% and was in line with globally reported standards (67). Finally, multiple testing might regard as a weakness of retrospective data analyses.

In conclusion, in addition to the stage- and entity-dependent cancer prognosis, the prognostic impact of RBC transfusions was detected only in patients with EC. In OC patients, the preoperative determination as “G8-frail” was associated with an independent worse oncological outcome. The different impact of RBC transfusions concerning the cancer entity could be firstly explained by the fact that EC patients in general were less likely to be as frail as OC patients. Secondly, the 5-year OS in the non-transfused OC cohort was fundamentally lower than in the EC cohort (46.3% vs. 82.6%). This survival disadvantage of the OC patient seemed to be explained by the fact that the diseases at diagnosis were more advanced, and the tumor biology, in general, seemed to be more aggressive. A standardized AOC clarification, as well as an evidence-based screening of frailty status, might be established in a preoperative diagnostic pathway to improve the individual cancer prognosis. However, due to the abovementioned limitations, a multi-centric or even prospective approach might be helpful to elucidate further information on our goal to improve the perioperative workup of cancer patients.

## Data availability statement

The original contributions presented in the study are included in the article/supplementary material. Further inquiries can be directed to the corresponding author.

## Ethics statement

Ethical review and approval was not required for the study on human participants in accordance with the local legislation and institutional requirements. The patients/participants provided their written informed consent to participate in this study.

## Author contributions

KA, and MB conceived and designed the study. KA, MWS, AL, AH, VL, WW, CW, SK, RS, MS, RR, EH, WB and MB collected the data. KA, MWS and MB performed the statistical analysis. KA and MB wrote the manuscript. All authors critically revised the manuscript and contributed to the article and approved the submitted version.

## Conflict of interest

KA reports personal fees from Eisai, Roche, MSD. MWS reports holding a patent WO 2021/176091 A1 not related to this study. SK received speaker Honoria, research funding and travek reimbursement from Vovartis Pharma GmbH Germany. MS reports personal fees from AstraZeneca, BioNTech, Daiichi Sankyo, Eisai, Lilly, MSD, Novartis, Pantarhei Bioscience, Pfizer, Roche, and SeaGen outside the submitted work. Institutional research funding from AstraZeneca, BioNTech, Eisai, Genentech, German Breast Group, Novartis, Palleos, Pantarhei Bioscience, Pierre Fabre, and SeaGen. In addition, MS has a patent for EP 2390370 B1 and a patent for EP 2951317 B1issued. RS reports honoraria and expenses from Roche Pharma AG and AstraZeneca GmbH. AH reports honoraria and expenses from AstraZeneca, FBA Frauenärzte BundesAkademie GmbH, KlarigoVerlag, MedConcept, Med public GmbH, Med update GmbH, Medicultus, Pfizer, Promedicis GmbH, Pierre Fabre Pharma GmbH, Softconsult, Roche Pharma AG, Streamedup! GmbH, Tesaro Bio Germany GmbH. I am consultant to PharmaMar, Promedicis GmbH, Pierre Fabre Pharma GmbH, Roche Pharma AG and Tesaro Bio Germany GmbH. I have received funded research from Celgene. MB reports honoraria and expenses from Pharma Mar, Astra Zeneca, Tesaro, GSK, Roche, Clovis Oncology.

The remaining authors declare that the research was conducted in the absence of any commercial or financial relationships that could be construed as a potential conflict of interest.

## Publisher’s note

All claims expressed in this article are solely those of the authors and do not necessarily represent those of their affiliated organizations, or those of the publisher, the editors and the reviewers. Any product that may be evaluated in this article, or claim that may be made by its manufacturer, is not guaranteed or endorsed by the publisher.

## Abbreviations

AOC, Anemia of cancer; BMI, Body mass index; CGA, Comprehensive Geriatric Assessment; 95%-CI, 95%-confidence interval; DSS, disease-specific survival; EC, endometrial cancer; ECOG, Eastern Cooperative Oncology Group; EQUATOR, Enhancing the QUAlity and Transparency Of health Research; FIGO, International Federation of Gynecology and Obstetrics; G8 **s**core, G8 geriatric Screening tool; HR, hazard ratio; MNA, Mini Nutritional Assessment; OC, ovarian cancer; OS, overall survival; OR, odds ratio; PFS, progression free survival; RBC, red blood cell; SCS, Surgical Complexity Score; SIOG, International Society of Geriatric Oncology; STROBE, Strengthening the reporting of observational studies in epidemiology.
